# Low self-esteem predicts orthorexia nervosa, mediated by spiritual attitudes among frequent exercisers

**DOI:** 10.1007/s40519-020-01095-z

**Published:** 2021-01-27

**Authors:** Enikő Bóna, Anett Erdész, Ferenc Túry

**Affiliations:** 1 Institute of Behavioural Sciences, Semmelweis University, Budapest, Hungary; 2 Faculty of Education and Psychology, Eötvös Loránd University, Budapest, Hungary

**Keywords:** Orthorexia nervosa, Self-esteem, Mediation analysis

## Abstract

**Objective:**

The aim of this study was to measure how orthorexic tendencies relate to age, self-esteem, and spirituality. We conducted the study on a sample of Hungarian adults performing regular fitness activity.

**Method:**

175 participants completed a four-part online survey: demographics and training habits, the Rosenberg Self-Esteem Scale, the Eating Habits Questionnaire-Revised (EHQ-R), and one of the Spiritual Awareness questionnaire’s subscale. We performed univariate linear regression to assess the predictor role of age on orthorexic tendencies. Mediation analysis was conducted to determine the effect of self-esteem on orthorexic tendencies and the mediator role of spiritual attitudes.

**Results:**

Age negatively correlated with EHQ-R, and there were no gender differences. Lower self-esteem was a predictor for orthorexic tendencies with the total effect of *ß* = -0.3046 (*p* < 0.0001). In part, this is a direct relationship, but it is mediated by spiritual awareness as well.

**Discussion:**

Among frequent exercisers, strict dieting is likely to originate from a lack of self-esteem due to perfectionist standards, social comparison, and the aspiration of being in control. In case self-esteem is achieved through spiritual approaches, individuals may experience positive changes in their attitudes toward eating and their bodies as well. In the future, it is important to confirm whether the EHQ-R indicates diagnostic boundaries.

**Level of evidence:**

Level V, descriptive cross-sectional study.

## Introduction

Orthorexia nervosa (ON) is known as a form of disordered eating by rigidly adhering to different healthy diets and eventually becoming dependent on these habits. The main psychological correlates of ON are perfectionism and obsessive–compulsive disorder [1–4]; moreover, anorexia nervosa and bulimia nervosa can exist in the presence of ON tendencies [5–7]. A recent case study also demonstrated that malnutrition aggravates the severity of depression and anxiety [8]. A narrative review conducted by the Orthorexia Nervosa Task Force in 2018 summarized the diagnostic proposals that have been published so far and attempted to set up the boundaries of ON definitions. Their results show that the following indications are present on all lists: obsessional or pathological preoccupation with healthy nutrition that escalate over time, emotional consequences (e.g., distress, anxieties) of non-adherence to self-imposed nutritional rules, and finally, psychosocial impairments in relevant areas of life as well as malnutrition and weight loss [9].

Communities that are centered around exercising and fitness deserve special attention while examining eating disorders and ON. Although exercising and healthy eating are both valuable and essential components of wellbeing, trends of the health and wellness industries may potentially be harmful when they are followed excessively, causing impairments in everyday life [10]. Since following such a lifestyle is socially accepted and encouraged, these dangers can remain unnoticed. Numerous studies examined whether those who practice excessive exercise are likely to develop eating disorders [11–14]. Even though the evidence is disputable, the theory that ON is more widespread among frequent exercisers has been partly validated [15, 16].

Besides being exposed to the messages of the health industry, younger age can also be a factor in developing ON in the population of fitness communities: several studies found that in such groups the younger the participants are, the more orthorexic tendencies they had [14, 17, 18]. According to our best knowledge, orthorexia research using special samples so far has focused on people from age groups closer to the generation of university students [7]; thus, characteristics of a wider age range are yet to be explored.

Self-esteem is conceptualized as a global, unidimensional, and relatively stable construct regarding the personal judgment of one’s own worth [19]. Bratman and Knight [19] theorized that an orthorexic person’s self-esteem is often tied to their adherence to the diet. Furthermore, they would feel a sense of superiority over others based on their eating practices which are the primary focus of their lives. A recent meta-analysis concluded that self-esteem difficulties are highly relevant in the treatment of anorexia nervosa (AN) and that transdiagnostic approaches regarding other eating disorders (such as ON) can be recommended [20]. Despite its relevance in disordered eating and other destructive health behaviors [21], self-esteem was generally found to be unrelated to ON [7, 22–24].

In the early history of eating disorders, there are sources that provide a spiritual explanation, such as, speaking of religious figures who lived ascetic lives, using fasting as a religious rite [25]. The lack of desires is central here that the term anorexis also refers to. Spirituality is a broader concept than religion and relates to a drive to search for transcendent meaning [26, 27]. The relationship between spirituality and ON can be evaluated from two aspects. The first is the pseudospiritual meaning of the dependence on healthy food, suggesting that the violation of self-imposed diet rules causes a sense of personal impurity, accompanied by compulsive thoughts and shame. This leads to penitence and even stricter dietary restrictions, which are regarded as clean eating and obsessive dieting [7, 28]. Creating these magical belief systems about food and health may serve as a link to “eating-disordered thinking” [27]. By contrast, the second aspect of spirituality is its protective role against disordered eating, serving as a therapeutic factor. In their study, Boisvert and Harrel [29] found that higher spirituality was related to a lower level of eating disorder symptomatology, while other studies support the positive effects of spiritual involvement on health behavior, as well [26, 30].

The connection between self-esteem, pursuit of spiritual purity, and ON may have a common ground in the compulsive nature of all three. For those who suffer from obsessive–compulsive behavior, eating related or not, low self-esteem is a general vulnerability factor [24, 31]. Thus, seeking spiritual purity via dieting by its compulsive nature [2] may increase the difficulties of obtaining self-esteem.

In this present study, we aim to find out how orthorexia nervosa (measured by using the Eating Habits Questionnaire—Revised) relates to age, self-esteem, and spirituality. Our sample consisted of Hungarian adults performing regular physical fitness activity. In our first hypothesis, in accordance with the previous literature, we expect to find a negative relationship between age and ON: we predict that the younger our participants are, the more orthorexic they are. Second, we hypothesize a link between self-esteem and ON, expecting that low self-esteem would predict orthorexic tendencies. Even though there is no such connection revealed yet, we decided to introduce this hypothesis due to its theoretical background and the close connection between AN and self-esteem [20] and , furthermore, OCD and self-esteem [24, 31]. Third, we assume that this connection is mediated by spirituality in a way that higher levels of spiritual attitudes would predict orthorexic tendencies.

## Method

This cross-sectional study was conducted between April and June 2019. We collected data from Hungarian adults who perform recreational exercise regularly (at least three times per week). Our questionnaire was primarily distributed online using the Google Forms application via social media posts, both in open and closed groups. Furthermore, it was also mailed to members of a fitness training college in Budapest. Altogether, 181 responses were collected from which we removed one invalid response, underage participants (*n* = 2), and individuals over 60 (*n* = 2). We decided to do so because those over 60 have an increased likelihood of chronic illnesses that may require special dietary management or dietary restrictions which may be associated with features similar to ON. We also excluded a 42-year-old woman who, according to her self-reported weight, was just 28 kg, leaving a final sample of 175 subjects (50 males and 125 females). The study was approved by the Hungarian Medical Research Council (Ethics Approval Number: TUKEB 3563-1). Anonymity was assured to all participants. All procedures performed in this study comply with The Code of Ethics of the World Medical Association (Declaration of Helsinki) for experiments involving humans.

### Measures and variables

Our survey contained four sections. In the first section, respondents were asked about their gender, age, education, and place of residence. Their body mass index (BMI) was calculated from self-reported weight and height. This section also included their information about training habits: we asked how long they have been training (0–5 years, 6–10 years, more than 11 years) and how frequently they do so. The frequency of “at least three times per week” was already prerequisite at the start of the questionnaire (it was mentioned in the survey’s introduction), but we also added “daily” and “multiple times a day” responses.

In the second section, participants completed the Hungarian version of the Eating Habits Questionnaire—Revised [32]. The original Eating Habits Questionnaire (EHQ) [33], a 21-item instrument, considers three aspects of ON (knowledge of healthy eating, problems associated with healthy eating, and feeling positively about healthy eating). This altered, 30-item version of the EHQ was developed in 2018. In the process of its creation, we were in constant email communication with the author and decided to adapt the 30-item EHQ-R to Hungarian due to its more refined nature. The instrument has five subscales. “Rigidity” subscale assesses the compliance level to self-prescribed dietary rules, using seven items. The six questions of “Healthy body appearance” subscale are aimed to measure anxiety about superficial signifiers of health and how participants connect healthy external signs and outward appearance to dieting. The 7-item “Violation of dietary rules” focuses on how the individual can handle the consequences of violating their own dietary rules. The six items of the “Negative emotionality” dimension assess the presence of stress, anxiety, guilt, and shame which can occur after impure eating. Finally, “Time impairment” subscale aims to measure whether one devotes an excessive amount of time to the implementation of a healthy diet in four questions. The higher the global score, the more disordered the dietary rules are and when followed by the individual they are more distinguishable from adopting a balanced diet. Each of the 30 items is rated on a four-point Likert scale between “not at all” (1) and “completely true” (4). Scoring is completed by adding the items; there are no reversed questions in EHQ-R. The Cronbach’s alpha was calculated to test the inner reliability of this questionnaire, which ranged from 0.75 to 0.87, confirming the internal consistency of the subscales.

The third section listed the questions of the Rosenberg self-esteem scale (RSES) which is a one-dimensional, 10-item self-report instrument. Participants responded using a Likert scale ranging from 1 (“strongly disagree”) to 4 (“strongly agree”). For the present study, the RSES’s internal reliability was good (Cronbach’s alpha: 0.89).

Finally, the fourth part presented the one subscale of the Spiritual Awareness Scale [34]. The eight questions in this subscale aim to find out whether the participant has a healthy relationship with oneself and their surrounding environment, while having obtained a sense of spirituality. The person who scores high on this test is not reactive or oversensitive in their interpersonal relationships, does not rely on external sources of validation, and is not troubled by perfectionist aspirations. Instead, they are characterized by being calm and not feeling burdened with their daily activities. This inner peace is attributed to the decreased functioning of the “Ego”. The answers range on a 6-point Likert scale: Never or almost never (6), Rarely (5), Sometimes (4), On most days (3), Every day (2), and Multiple times a day (1). In the present study, internal reliability was good (Cronbach alpha: 0.85).

### Sample

Our final sample contained 175 participants (50 males and 125 females). Their average age was 35.4 years (SD = 7.59 years; range: 18–57 years). The average BMI was 24.8 (SD = 4.94, range: 17.4–47.8). Two percent of the participants finished primary, 20% finished secondary school, and 78% have a higher education diploma. 55% of the respondents had their place of residence in the capital city, 29% live in a big city, 9% live in a (large) village, and 7% is a countryside dweller.

### Data analyses

All analyses were performed with SPSS 21.0. To test for sex differences, we completed a chi-square test, a two-sample *t* test, and a Mann–Whitney *U* test in the case of non-normal distribution. We used Cohen's definition [35] to interpret the values of the correlation coefficients (weak under 0.3, moderate between 0.3 and 0.5, and strong over 0.5). We performed univariate linear regression to assess the predictor role of age on orthorexic tendencies.

**Fig. 1:**
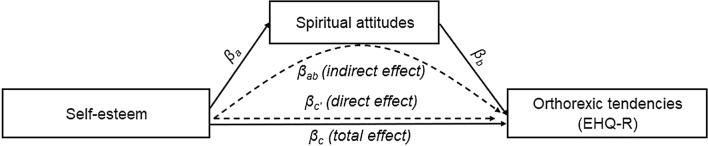
Mediation paths between self-esteem and orthorexic tendencies

Mediation analysis was conducted using the PROCESS macro (v3.4) [36] to determine the effect of spiritual awareness on the relationship between self-esteem and orthorexic tendencies. PROCESS calculates three different regressions, as Fig. 1 illustrates: the first is for measuring the total effect (the relationship between the predictor and the outcome variable: *β*_c_), the second is to measure the mediator variable’s effect (*β*_a_), and finally, the third model includes both the predictor and the mediator variable as well, and thus, the direct effect is calculated (*β*_c’_ and *β*_b_). PROCESS uses a bias-corrected bootstrap confidence interval approach when providing estimates of indirect effects (β_ab_). Mediation occurs when the significance of the direct effect is either no longer significant or the size of its effect is reduced in the presence of the mediator. We completed these analyses first using the summarized scores of EHQ-R as a dependent variable, then we checked the relationships with each dimension of ON by testing with each EHQ-R subscale.

## Results

### Sample description and relationship between gender and orthorexia

The mean score for EHQ-R was 59.39 (SD 16.57, range: 34–118). 44.6% of study participants have been training regularly for less than 5 years, 26.3% for 6–10 years, and 29.1% for at least 11 years. 89.7% of respondents train several times a week, 7.4% daily, and 2.9% train several times a day. There were no significant gender differences in orthorexic tendencies, although the Rigidity subscale presented a trend toward significance for women (*t* (173) = -1.807; *p* = 0.073) in our sample (Table 1).

**Table 1:** EHQ-R scores and gender differences

Scales	Men (*n* = 50)	Women (*n* = 125)	Comparison of the groups
	*M* (SD)	*M* (SD)	
EHQ-R summed scores	56 (15.51)	60 (16.91)	*t*(173) = − 1.373
Rigidity	13.6 (4.27)	14.9 (4.69)	*t*(173) = − 1.807^+^
Healthy body appearance	14.8 (3.72)	15.3 (3.76)	*t*(173) = − 0.772
Violation of dietary rules	11.8 (4.09)	12.6 (4.45)	*Z* = 1.268
Negative emotionality	9.7 (3.40)	10.5 (4.03)	*Z* = 1.302
Time impairment	6.8 (2.36)	7.1 (2.55)	*Z* = 0.672

^+^*p* < 0.100

The RSES’s mean score was 26.96 (SD 5.78, range: 11–40) and the subscale measuring spiritual awareness had a mean score of 33.26 (SD 7.81, range: 11–47).

### Relationship between age and orthorexia

According to the results of our analysis, age negatively correlated with EHQ-R (*r* = -0.150, *p* = 0.047). Regarding the subscales, negative emotionality (*r* = -0.18; *p* = 0.017) and time impairment (*r *= -0.21; *p* = 0.005) proved to be significant, while in the case of rigidity, violation of dietary rules, and healthy body appearance, there was no significant association found with age.

### Relationship between orthorexia, self-esteem, and spirituality

In our multiple regression model, low self-esteem predicted ON in a way that Rosenberg scale’s results had a negative effect on the summed scores of EHQ-R (the total effect: *ß*_c_ = -0.3046, *p* < 0.0001). While using a mediation analysis, it was shown that there was a significant direct effect (*ß*_c’_ = -0.1793, *p* < 0.0001) and indirect effect as well (*ß*_ab_ = -0.1271, LLCI: -0.2349, ULCI: -0.0416). Additionally, it can also be seen that ON tendencies depend on spiritual awareness (*ß*_b_ = -0.2960, *p* = 0.002).

Regarding the subscales, self-esteem had a significant negative total effect on all five scales of the EHQ-R: Rigidity, Healthy body appearance, Violation of dietary rules, Negative emotionality, and Time impairment. After testing for the mediator role of Spiritual awareness, it can be seen that self-esteem had no significant direct effect on Healthy body appearance and Time impairment, only an indirect effect through mediation. Violation of dietary rules and Negative emotionality have both immediate direct effect from self-esteem and mediated effect through Spiritual awareness. In the case of Rigidity, neither direct nor indirect effects were significantly present.

The details of our regressions can be found in Table 2.

**Table 2:** Results of mediation analysis—the effects of self-esteem on orthorexic tendencies mediated by spiritual attitudes

Output	Predictor	Path	*R*^2^	*F*	*Β*	se	*t*	*p*	LLCI	ULCI
Spirituality	Self-esteem	*β*_a_	0.1844	39.1156	0.4294	0.0687	6.2542	< 0.0001	0.2939	0.5649
EHQ-R	Self-esteem	*β*_c_ (total effect)	0.0939	17.9292	−0.3064	0.0724	−4.2343	< 0.0001	−0.4493	−0.1636
	Self-esteem	*β*_c′_ (direct effect)	0.1654	17.0399	−0.1793	0.0771	−2.3248	0.0213	−0.3316	−0.0271
	Spirituality	*β*_b_	0.1654	17.0399	−0.2960	0.0771	−3.8377	0.0002	−0.4483	−0.1438
	Self-esteem	*β*_ab_ (indirect effect)	–	–	−0.1271	0.0495	–	–	−0.2349	−0.0416
Rigidity	Self-esteem	*β*_c_	0.0405	7.3086	−0.2013	0.0745	−2.7034	0.0075	−0.3483	−0.0543
	Self-esteem	*β*_c′_	0.0594	5.4329	−0.1360	0.0819	−1.6607	0.0986	−0.2976	−0.0256
	Spirituality	*β*_b_	0.0594	5.4329	−0.1522	0.0819	−1.8584	0.0648	−0.3138	−0.0095
	Self-esteem	*β*_ab_	–	–	−0.0653	0.0448	–	–	−0.1623	−0.0117
Violation of dietary rules	Self-esteem	*β*_c_	0.0834	15.7461	−0.2888	0.0728	−3.9681	< 0.0001	−0.4325	−0.1452
	Self-esteem	*β*_c′_	0.1562	15.9212	−0.1605	−0.0776	−2.0701	0.0399	−0.3136	−0.0075
	Spirituality	*β*_b_	0.1562	15.9212	−0.2987	−0.0776	−3.8519	0.0002	−0.4518	−0.1457
	Self-esteem	*β*_ab_	–	–	−0.1283	0.0474	–	–	−0.2304	−0.0454
Negative emotionality	Self-esteem	*β*_c_	0.1501	30.5559	−0.3874	0.0701	−5.5277	< 0.0001	−0.5258	−0.2491
	Self-esteem	*β*_c′_	0.2131	23.2864	−0.2681	0.0749	−3.5799	0.0004	−0.4160	−0.1203
	Spirituality	*β*_b_	0.2131	23.2864	−0.2779	0.0749	−3.7098	0.0003	−0.4257	−0.1300
	Self-esteem	*β*_ab_	–	–	−0.1193	0.0481	–	–	−0.2252	−0.0370
Body image	Self-esteem	*β*_c_	0.0530	9.6788	−0.2302	0.0740	−3.1111	0.0022	−0.3762	−0.0841
	Self-esteem	*β*_c′_	0.1086	10.4827	−0.1180	0.0797	−1.4802	0.1406	−0.2753	0.0393
	Spirituality	*β*_b_	0.1086	10.4827	−0.2613	0.0797	−3.2774	0.0013	−0.4186	−0.1039
	Self-esteem	*β*_ab_	–	–	−0.1122	0.0446	–	–	−0.2016	−0.0369
Time impairment	Self-esteem	*β*_c_	0.0456	8.2617	−0.2135	0.0743	−2.8743	0.0046	−0.3601	−0.0669
	Self-esteem	*β*_c′_	0.1405	14.0547	−0.0670	0.0783	−0.8561	0.9391	−0.2215	−0.0875
	Spirituality	*β*_b_	0.1405	14.0547	−0.3411	0.0783	−4.3576	< 0.0001	−0.4956	−0.1866
	Self-esteem	*β*_ab_	–	–	−0.1465	0.0454	–	–	−0.2433	−0.0667

## Discussion

Results indicate that our sample of young adults who exercise regularly have a normal BMI on average, they are predominantly college educated, and live in the capital city. They have scored in the middle range on the EHQ-R test. We found no significant difference between males and females. These findings are in line with the results of past studies conducted on samples with the same characteristics (i.e., performing fitness activity regularly), but using different questionnaires [17, 37]. This result suggests that ON is not typically overrepresented in fitness communities [17]; furthermore, it is expected to stay unrelated to gender, irrespective of what tool was being used.

### Age

We hypothesized younger age to be associated with ON in our sample. From the results, it appears that younger participants had more orthorexic tendencies. This relationship has been confirmed in the context of anorexic and bulimic eating behavior [38], and our finding also echoes recently published research that use the special sample of fitness participants [14, 17, 18]. The same relationship was exposed in studies that measured ON in a student population: Croatian adolescents [39], Turkish medical students [40], and Italian university students of mixed majors [41]. The present study can be considered a rare one among its kind operating with the average age of 35.4. More studies are necessary to explore whether this directionality is also present among middle-aged and older adults. Also, future exploratory research should identify the reason why lower age makes one more vulnerable to orthorexic practices.

### Self-esteem and orthorexic tendencies

According to our data, self-esteem is a predictor for ON, in a way that higher scores of EHQ-R were associated negatively with the RSES results. This significant relationship can be seen in the case of all five subscales of EHQ-R. Despite being aware that no published research has indicated such links [7], we decided to test our hypothesis based on both academic and non-academic scientific literature which introduces certain theories that our results did end up supporting. When making a connection between self-esteem and ON, the handbook “Orthorexia: When Healthy Eating Goes Bad” written by Renee McGregor, a registered dietitian, must be acknowledged. The author describes behavioral patterns she has observed as a practitioner on her clientele, explaining orthorexic tendencies originating from people pushing their potentials to their limits in order to gain self-esteem. Her notes and observations in the handbook point out that ON is rather about obsessive self-development than only achieving a perfect body weight [42]. The possible origins of orthorexic tendencies are explained that striving for a perfect diet may lead to higher sense of self-worth, as those who follow special diets may feel virtue due to their sometimes extreme eating behaviors [43]. The constant struggle to “become better” has been mentioned in recent qualitative ON-related studies as well. Greville-Harris et al. analyzed social media blog expressions of ON which described their stubborn diet adherence as a result of critical self-talk and a wish to fulfill unrelenting perfectionist standards. The bloggers also admitted having a sense of superiority and applying downward comparisons, identifying themselves as better and healthier than others [28]. This is in line with the literature on social comparison as those having low self-esteem are likely to engage in these behaviors and may have their satisfaction increased after the downward comparison [44].

The relationship in our data between low self-esteem and orthorexic tendencies may be explained by existential fear deriving from societal pressures. It has been described that it is socially accepted to be strict and controlled by diet trends because having a disciplined and conscious attitude is a marker of health consciousness [45]. It is common knowledge that adhering to a healthy diet is a great protective factor against obesity, diabetes and other metabolic disorders. However, from the terror management theory aspect, people are driven to live up to cultural standards of health values [46], as awareness of their own mortality motivates them to obtain higher self-esteem [47]. This theory suggests that these health behaviors might go to the extent of entailing health risks (that is, excessive dieting). Thus, they may behave in ways that could compromise their physical health to garner security from existential concerns about mortality. It has been shown that low self-esteem is one of the most prominent risk factors for developing DSM-5 eating disorders [48]. Identifying low self-esteem, perfectionist attitudes and excessive self-development in daily common health practices are important as these may serve as possible risk factors in developing ON, even though at first sight, they might seem as socially accepted health trends.

### Spiritual awareness as a mediator

As seen, the higher the self-esteem was for our participants, the less likely orthorexic tendencies were present in their eating behavior. This relationship was mediated through spiritual awareness. This contradicts our hypothesis that predicted the opposite relationship: it was thought that food rituals and the exaggerated faith in the healing quality of certain foods would resemble religious and spiritual belief systems. We suggested that stronger spiritual approaches would mean higher orthorexic tendencies, and despite the empirical data [29] that had supported these theories [26, 30], our results demonstrated that the higher the spiritual awareness was among our participants, the less likely it was that they were orthorexic.

Negative emotions and guilt about breaking dietary rules are influenced by self-esteem, both directly and indirectly (mediated through spirituality). Worrying about a healthy body appearance and being impaired by time-consuming rituals are affected by self-esteem, but only through the mediating role of spiritual awareness.

Those who score high on the subscale of the Spiritual Awareness Scale, are not concerned about what others think about them and do not long for more than they already have [34], and this predicted a negative tendency on the Healthy body appearance scale. This suggests that the higher one’s self-esteem is due to this peaceful approach, the less likely it is that they depend on an uncomfortable desire to appear healthy, or the extrinsic validation about their looks. Both qualitative and quantitative data support that those who experience this sense of spirituality and contentedness, experience positive changes in attitudes toward eating and their body image as well [49]. Similarly, EHQ-R’s Time impairment subscale directly might not be predicted by low self-esteem, but the lack spiritual awareness could become an important link to the time-consuming, burdening habits. A possible explanation to this mediating effect is that spiritual practices may lead to increased acceptance and less need for rigid discipline and planning. This association has been shown among yoga practitioners reporting that they could focus on the present more effectively which is important when freeing themselves from self-imposed controlling behaviors [49]. This attitude helps the recovery from eating disorders, especially for those with a drive for spending uncomfortable amounts of time on food planning. The helpful effect of spiritual practices is supported by the data that Hall and Cohn [50] collected. They interviewed 366 women and 6 men about the activities that helped them recover most from bulimia nervosa and other types of eating disorders. 58% of the respondents reported that spiritual activities (e.g., prayer, meditation, etc.) were useful.

Even though disordered eating was associated with spiritual practices in past research, we can conclude that several sources support our findings claiming that spiritual-religious content may not only be destructive, but also supportive, promoting healing. Berrett et al. [51] summarized the relationships among trauma, eating disorders and spirituality. From a therapeutic point of view, both trauma and eating disorder can distance patients from their spirituality, viewed as a treatment resource. Clinicians should assess the spirituality levels of patients in their lives to help create an individualized treatment plan. It can be an important therapeutic task to help patients access the resources of their spirituality. Spiritual discussions and interventions may help patients with eating disorders.

### Limitations

Since the study was performed on a sample with special characteristics, one of its limitations is that it cannot be generalized to those who do not perform regular fitness activity. Furthermore, females, participants with higher education and capital residents were overrepresented which also limits the generalizability of our results. A further limitation is that we are not currently aware of EHQ-R’s critical cutoff score. Due to the lack of clarity around the diagnostic boundaries, higher scores do not imply automatically that the displayed eating behavior is harmful. In the future, it is important to confirm with further studies that the EHQ-R is a reliable measuring or diagnostic tool. Conducting fitted control group studies may help determine the cutoff points.

## Conclusion

This study was the first to investigate self-esteem and spirituality as they relate to orthorexic tendencies. Our findings support that in the community of frequent exercisers, for both genders, primarily among younger individuals, mental healthcare is necessary, focusing on self-esteem by lowering perfectionism and the need for control. Higher self-esteem may be achieved through spiritual practices (e.g., yoga and practicing body awareness); thus, it is possible to reduce the incidence of ON and the occurrence of complications.

## What is already known on the subject?

Several studies have investigated the presence of ON in special samples such as fitness communities. The evidence is not consistent in relation with detecting the association between frequent exercising and orthorexic eating behaviors; however, it has been shown that it is comorbid with anorexia nervosa and negatively correlates with age and positively with exercise frequency. The constructs of self-esteem and spirituality have rarely been examined in connection with ON, and those who studied it did not conclude any significant results, or the studies conducted were taking DSM eating disorders into consideration and not ON.

## What does this study add?

It is concluded that spiritual approaches could have a beneficiary effect in gaining self-esteem and thus improving orthorexic eating practices that have emerged among fitness communities. Our aim is that this paper would add to the inventory of studies that may be useful for decision makers in the health and wellness industry. It is proposed to help those fitness enthusiasts who might develop orthorexic eating habits due to their lack of self-esteem and aspire for perfection and self-development. Studies are necessary to investigate the type of spiritual practices or psychotherapies that might help those who fall into the ON category.

## Data Availability

The pull of data that supports the findings of this study is available with the corresponding author and provided upon reasonable request.
